# Genomic Diversity of CRESS DNA Viruses in the Eukaryotic Virome of Swine Feces

**DOI:** 10.3390/microorganisms9071426

**Published:** 2021-07-01

**Authors:** Enikő Fehér, Eszter Mihalov-Kovács, Eszter Kaszab, Yashpal S. Malik, Szilvia Marton, Krisztián Bányai

**Affiliations:** 1 Veterinary Medical Research Institute, Hungária Krt 21, H-1143 Budapest, Hungary; feher.eniko@vmri.hu (E.F.); kovacsesztermail@gmail.com (E.M.-K.); kaszab.eszter@vmri.hu (E.K.); marton.szilvia@vmri.hu (S.M.); 2 College of Animal Biotechnology, Guru Angad Dev Veterinary and Animal Sciences University, Ludhiana 141004, Punjab, India; malikyps@gmail.com; 3 Department of Pharmacology and Toxicology, University of Veterinary Medical Research, István Utca. 2, H-1078 Budapest, Hungary

**Keywords:** CRESS DNA virus, swine, metagenome, phylogenetic analysis, Hungary

## Abstract

Replication-associated protein (Rep)-encoding single-stranded DNA (CRESS DNA) viruses are a diverse group of viruses, and their persistence in the environment has been studied for over a decade. However, the persistence of CRESS DNA viruses in herds of domestic animals has, in some cases, serious economic consequence. In this study, we describe the diversity of CRESS DNA viruses identified during the metagenomics analysis of fecal samples collected from a single swine herd with apparently healthy animals. A total of nine genome sequences were assembled and classified into two different groups (CRESSV1 and CRESSV2) of the *Cirlivirales* order (*Cressdnaviricota* phylum). The novel CRESS DNA viral sequences shared 85.8–96.8% and 38.1–94.3% amino acid sequence identities for the Rep and putative capsid protein sequences compared to their respective counterparts with extant GenBank record. Data presented here show evidence for simultaneous infection of swine herds with multiple novel CRESS DNA viruses, including po-circo-like viruses and fur seal feces-associated circular DNA viruses. Given that viral genomes with similar sequence and structure have been detected in swine fecal viromes from independent studies, investigation of the association between presence of CRESS DNA viruses and swine health conditions seems to be justified.

## 1. Introduction

Circular Rep-encoding (replication-associated protein encoding) single-stranded DNA (CRESS DNA) viruses form a highly diverse group of small viruses that have been detected worldwide in prokaryotes and eukaryotes, as well as in environmental samples [1,2]. At present, the classified eukaryotic CRESS DNA viruses belong to seven virus families of the *Cressdnaviricota* phylum (*Shotokuvirae* kingdom, *Monodnaviria* realm), including *Bacilladnaviridae*, *Nanoviridae*, *Smacoviridae*, *Geminiviridae*, *Genomoviridae*, *Redondoviridae,* and *Circoviride* [3]. The genome of CRESS DNA viruses identified in eukaryotic organisms is typically 1–6 kilobases long and contains two main ORFs encoding the Rep and the capsid (Cp) proteins in variable orientation [2,3,4]. In addition, a number of other CRESS DNA viruses could not be classified yet, and the diversity of the genomic sequences poses challenges to experts in the field of virus taxonomy. Although *rep* is conserved and may be distinctive for variable CRESS DNA viruses, description of endogenous *rep*-like elements in eukaryote genomes draws attention to the appropriate selection of methods and usage of complete genome sequences for the classification of these viruses [5,6].

Members of the *Circoviridae*, *Smacoviridae*, and *Redondoviridae* family have been characterized as animal-associated viruses [3,7,8]. Although these families include a continuously expanding number of viruses, only a few of those have been proved to be pathogenic. One of the known porcine circoviruses, *Porcine circovirus 2*, is associated with multisystemic diseases in swine, posing threat to swine herds and losses for the breeders [9]. Besides that, both classified and unclassified CRESS DNA viruses have been identified in fecal samples of both healthy and diseased pigs [9,10,11,12,13,14,15,16,17,18,19,20,21]. The microbial community of the swine intestine is highly complex, and, as of now, very little is known about how the viruses, including these non-classified porcine-associated CRESS DNA viruses, interact with host cells and other microbes. In this study, we examined the occurrence of CRESS DNA viruses in porcine fecal specimens originally collected for metagenomic analysis of eukaryotic viruses. Representative genomes of novel viruses that showed similarities with porcine circovirus-like (po-circo-like) viruses and fur seal feces-associated circular DNA viruses were sequenced and characterized [16,17]. The study confirms high occurrence of diverse CRESS DNA viruses in the swine feces.

## 2. Materials and Methods

### 2.1. Sample Processing and Random PCR for Metagenomics

Metagenomic analysis was conducted from fecal samples of 23 swine (piglets *n* = 5, weaned pigs *n* = 8, fattening pigs *n* = 6, and breeding swine *n* = 4) collected in a farm in Tiszavasvári, Hungary in 2012. Fifty to one hundred mg feces were homogenized in 1 mL phosphate-buffered saline and centrifuged for 10,000× *g* for 5 min. Nucleic acid was extracted from the supernatant with Direct-zol RNA Miniprep kit (Zymo Research, Irvine, CA, United States) omitting DNase treatment. Five μL nucleic acid was denatured at 95 °C for 5 min, after addition of 1 μL of 25 μM FR26RV-N anchored random hexamer oligonucleotide, and was reverse transcribed [22]. The 25 μL reverse transcription reaction mixture contained the denaturation mixture, 400 μM of dNTP, 1x AMV RT Buffer, and 1U of AMV Reverse Transcriptase (Promega, Madison, WI, United States), and was incubated at 25 °C for 10 min, 42 °C for 60 min, and 70 °C for 15 min. Randomized amplification was carried out in 25 μL PCR mixture containing 400 μM of dNTP, 1 μM of FR20RV primer, 1x DreamTaq Buffer, 2.5 U of DreamTaq DNA Polymerase (Thermo Scientific, Waltham, MA, United States), and 3 μL of the cDNA. The reaction conditions consisted of an initial denaturation step at 95 °C for 3 min, 40 cycles of amplification with the steps of 95 °C for 30 s, 48 °C for 30 s, and 72 °C for 2 min, followed by a final elongation step at 72 °C for 8 min.

### 2.2. Sequencing

The amplified nucleic acid was subjected to DNA library preparation and sequenced with the Ion Torrent Personal Genome Machine™ PGM System (Thermo Fisher Scientific, Waltham, MA, USA). Enzymatic fragmentation and adapter ligation of the PCR products was performed with NEBNext® Fast DNA Fragmentation & Library Prep Set for Ion Torrent™ (New England Biolabs, New England Biolabs, Hitchin, UK) and the Ion Xpress™ Barcode Adapters kit (Thermo Fisher Scientific, Waltham, MA, USA). The barcoded samples were purified with Geneaid Gel/PCR DNA Fragments Extraction Kit (Geneaid Biotech, Taipei, Taiwan), and products between 300 and 350 bp were retrieved from 2% precast gel (Thermo Fisher Scientific, Waltham, MA, USA). The fragments were amplified using the reagents of the NEBNext® Fast DNA Fragmentation & Library Prep Set for Ion Torrent kit (New England Biolabs, New England Biolabs, Hitchin, UK). The steps of the procedure were initial denaturation at 98 °C for 30 s, followed by 12 amplification cycles at 98 °C for 10 s, 58 °C for 30 s, and 72 °C for 30 s, and final elongation at 72 °C for 5 min. The amplified library DNA was purified from agarose gel and was quantified with Qubit® 2.0 Fluorometer using Qubit™ dsDNA BR Assay Kit (Thermo Fisher Scientific, Waltham, MA, USA). Emulsion PCR of the mixed products were processed using Ion PGM™ Template Kit and OneTouch™ v2 instrument (Thermo Fisher Scientific, Waltham, MA, USA) according to the instructions. Templated bead enrichment with Ion OneTouch™ ES machine was performed according to the 200 bp sequencing protocol (Thermo Fisher Scientific, Waltham, MA, USA). The sequencing was carried out using Ion PGM™ Sequencing Kit on a 316 chip.

### 2.3. Amplification Of Complete Viral Sequences

The complete genome of CRESS DNA viruses was amplified with PCR in mixtures containing 200 nM of each primer (Table 1), 200 μM of dNTP mix, 1x Phusion Green buffer, 0.3 U of Phusion DNA Polymerase (Thermo Fisher Scientific, Waltham, MA, USA), and 2 μL nucleic acid. The cycling protocol consisted of a denaturation step at 98 °C for 30 s, 45 cycles of amplification with the steps of 98 °C for 10 s, primer annealing (temperatures in Table 1) for 30 s and 72 °C for 2 min, followed by a final extension step at 72 °C for 10 min. The PCR products were purified from agarose gel (Geneaid Gel/PCR DNA Fragments Extraction Kit, Geneaid Biotech, Taipei, Taiwan) and were subjected to library preparation and sequencing using the protocol described above.

### 2.4. Software

For viral metagenomics, raw sequence reads were trimmed and quality-controlled using CLC Genomics Workbench (version 9.0; 27/02/2016; http://www.clcbio.com). The minimal read length parameter was set to 35. Trimmed reads were taxonomically binned using Diamond v0.8.3 versus NCBI-NR [23]. After classification, the output files were analyzed and visualized by MEGAN6 Ultimate Edition [24].

The complete CRESS DNA viral genomes were assembled with Geneious Prime® 2020.2.4 software (https://www.geneious.com/, accessed on 16 September 2020) with mapping to references and *de novo* assembly of the reads. Coding sequences were predicted using the ORF Finder software (https://www.ncbi.nlm.nih.gov/orffinder, accessed on 16 September 2020) and putative ORFs encoding proteins of ≥100 aa were taken into consideration. Sequence alignments (using MAFFT algorithm) and pairwise sequence identities (using complete deletion option) were generated with Geneious Prime® 2020.2.4 and Mega6 software, respectively. Amino acid sequence-based maximum likelihood and neighbor joining trees were obtained with the PhyML and the Mega6 software, respectively, with the best fit models [25,26]. SH-like (maximum likelihood tree) and bootstrap (neighbor joining tree) supports were used to estimate the confidence of tree topology. Recombination analysis was performed with the Recombination Detection Program v4 [27].

## 3. Results

### 3.1. Results of Viral Metagenomics and Focus on CRESS DNA Viruses

**Figure 1:**
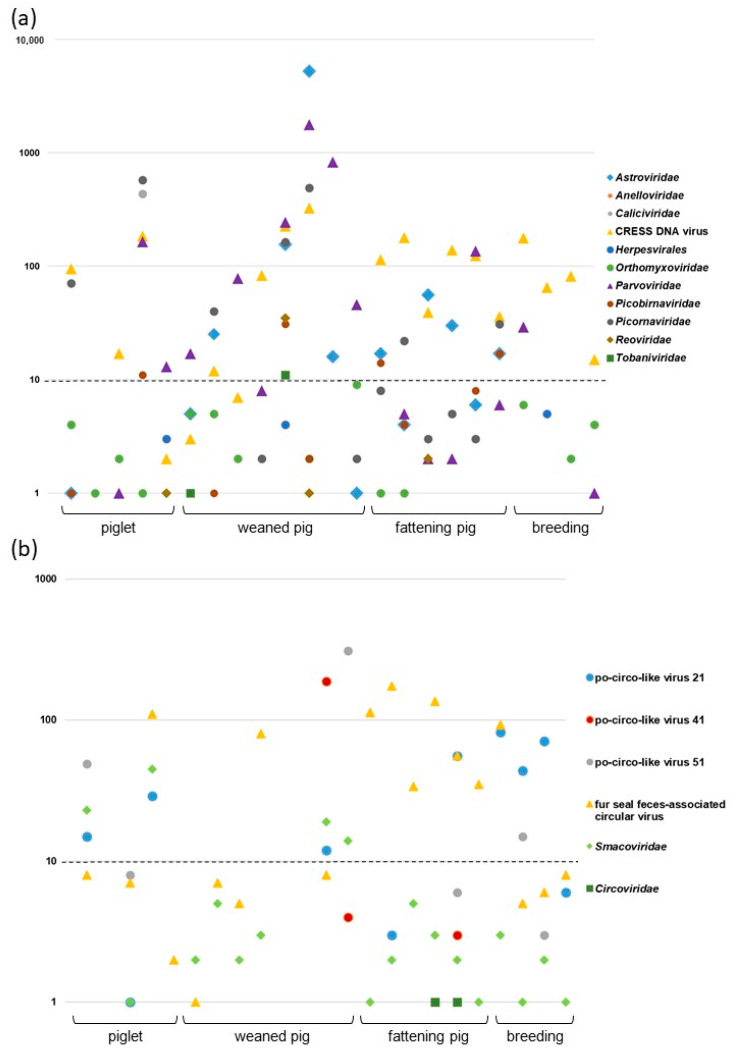
Virus families (**a**) and CRESS DNA viruses (**b**) represented in the fecal virome of swine investigated in this study. The read number for each viral group (Y axis) is plotted against the individual specimens sorted by age group. Symbols of viral taxa on the right are used in the plot. Detection of viral taxa represented by low read numbers (n < 10; dashed line) should be considered with cautions due to possible misidentification.

Metagenomic sequencing was conducted on 23 fecal samples collected from a swine farm in Hungary. Bioinformatic analysis of Ion Torrent reads identified a range of 2 to 7857 sequence reads per sample that mapped to viruses of eukaryotic organisms. These viral sequences could be classified into 12 virus families or orders (*Astroviridae*, *Anelloviridae*, *Caliciviridae*, *Cirlivirales*, *Herpesvirales*, *Orthomyxoviridae*, *Parvoviridae*, *Picobirnaviridae*, *Picornaviridae*, *Reoviridae*, *Smacoviridae*, *Tobaniviridae*; Figure 1a). All 23 samples contained viral sequences, and the number of identified viral families/orders ranged from 2 to 15 per sample.

The most genetically diverse virus group observed was CRESS DNA viruses that included the members of the *Cirlivirales* order and the *Smacoviridae* family. All additional analyses were conducted to characterize these viruses (Figure 1b).

Metagenomic analysis mapped numerous sequence reads to the genomes of CRESS DNA viruses in nearly all (20/23) samples (Figure 1). Based on BLAST search, the vast majority of reads (1–312 reads per virus per sample) in these 20 samples filtered for CRESS DNA viruses mapped to the sequences of porcine circovirus-like viruses (po-circo-like virus 21 and 22, 41 and 51, GenBank accession numbers: JF713716–719) and fur seal feces-associated circular DNA viruses (FSfaCVs, GenBank accession numbers: KF246569, LC133373, MK462122) [14,16,17,19,28].

Besides the reads associated with po-circo-like viruses and FSfaCVs, only a few reads (1 to 45 per sample) aligned with other known CRESS DNA viruses with up to 93% nt identities. The BLAST revealed that the reads fitted to sequences of strains from the *Smacoviridae* family. In addition to these viruses, two samples contained *Porcine circovirus 2* origin sequences, although at low quantity (a single sequence read in each), which raises the question of whether these sequence reads were true hits (Figure 1). In this study, we did not analyze these additional (putative) CRESS DNA virus sequences.

To determine the whole genome sequence of selected CRESS DNA viruses that were predicted to represent new viral variants, four back-to-back primer sets were designed for the amplification of complete viral genome sequences. Altogether, 15 PCR products of nine fecal samples were subjected to next-generation sequencing. Of these, nine amplicons from six fecal samples could be assembled to a complete genome; sequence ambiguities were seen in six long PCR products implying a mixture of highly similar sequences. Regarding the sequencing results, 17,075 to 28,428 sequence reads mapped to the assembled genomes with an average sequencing depth of 1178X (range, 635X to 1581X). Results indicated that the majority of sequences belonged to po-circo-like CRESS DNA viruses, while a minority of sequences were variant FSfaCVs.

### 3.2. Genomic Characterization of Novel Po-Circo-Like CRESS DNA Viruses

**Figure 2:**
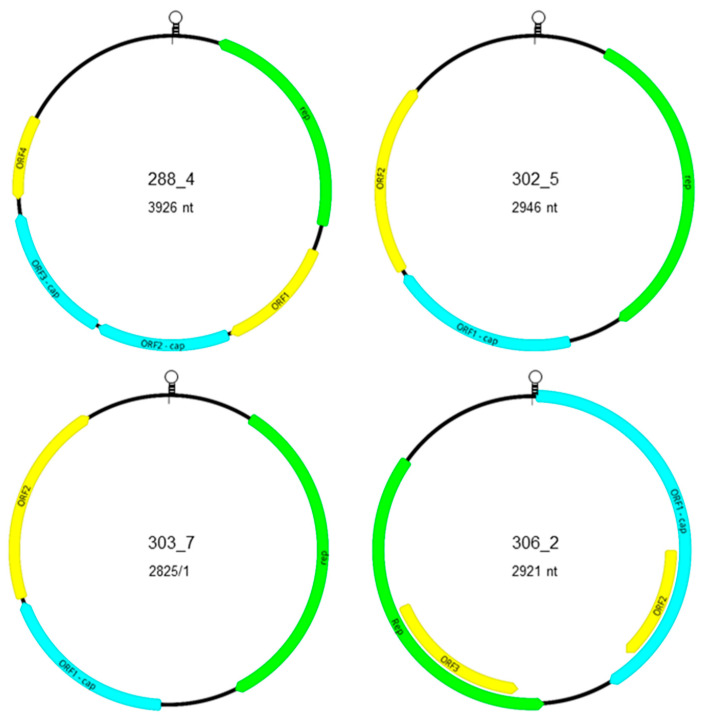
Genome arrangement and putative ORFs of the virus strains described in this study. The loop represents the nonanucleotide motif in the large intergenic region. The colored arrows show the direction and localization of the putative ORFs in the genome: green—*rep*; blue—*cp*; yellow—ORFs with unknown function.

The longest characterized genomes of 3926 bp belonging to the strains 288_4 and 302_4 showed 99.3% genome-wide nt identity with each other. The genome of the novel strains shared 85.3–87.7% identity with po-circo-like virus 21 and 22, GX14, GX15, and GX19 (GenBank Acc. no. MN263296–298), and 80.2% identity with the genome of the bovine origin bo-circo-like virus (GenBank Acc. no. MH316857) [16,19,29]. The predicted ORFs of the genome of strain 288_4 and 302_4 represented ambisense orientation and, using the conserved nonanucleotide motif for the determination of gene orientation, the *rep* may be located on the complementary strand of the replicative dsDNA intermediate (Figure 2, Table 1). According to the classification of Rosario et al. [4], the genome of these strains share key features with type IV CRESS DNA viruses.

The genome of strains 288_4 and 302_4 comprises a minimum of five ORFs, three in sense and two in antisense orientation (Figure 2; Table 2). The predicted *rep* and other ORFs were also identified in the porcine origin reference sequences with some differences, e.g., ORF3 was split into two smaller ORFs because of an early stop codon in the genome of po-circo-like virus 21. The length and sequence of the ORFs of bo-circo-like virus genome represented more differences compared to that of the porcine origin strains. The *rep* sequences of strain 288_4 and 302_4 showed 99.7% nt and aa identities with each other, 92.2–93.1 % nt and 96.1–96.8% aa identities with the *rep* sequence of po-circo-like virus 21, GX14, GX15, and GX19, and 87.7–89.6 % nt and 91.6–93.9% aa identities with the same region of po-circo-like virus 22 and bo-circo-like virus. Other ORFs of strain 288_4 and 302_4 showed 96.1–100% nt and aa identities with each other. The ORF1 and ORF4 of the strain 288_4 and 302_4 showed >90% nt and aa identity with the porcine origin reference sequences, while these values were lower for the ORF2 and ORF3 (75.9–86.3 nt and 74.4–85.6% aa identity for ORF2, and 49.9–66.8% nt and 38.1–54.5% aa identity for ORF3). Despite the sequence variability and lower identities, the N-terminal region of the ORF2 and ORF3 is relatively conserved, and slight accumulation of positively charged aa (R and K) in the derived protein sequences suggest that the gene products may be capsid proteins. The large intergenic region (LIR) was located between the 3’ end of the *rep* and 5’ end of ORF4 in the genome of strain 288_4 and 302_4. The LIRs of these sequences showed 100% nt identity with each other, and 91.8–95.2% nt identities with the porcine origin reference sequences. The putative nonanucleotide motif (CAGTATTAC) and the encompassing eight nt long inverted repeats is predicted to form a loop structure. Upstream of the nonanucleotide motif four copies of 10 nt long consecutive repeats were found as well. However, inverted repeats were missing that may indicate the lack of loop formation and thus may not be involved in the initiation of genome replication processes.

The predicted ORFs of the genome of strain 302_5, 303_5, 453_5, 303_7, and 453_7 showed unidirectional organization. The putative nonanucleotide motif and ORFs are located on the viral strand as described for type V CRESS DNA genomes [4] (Figure 2, Table 1). The nonanucleotide motif located in the LIR between the ORF2 and the *rep*, together with flanking inverted repeats, suggesting loop formation. The putative ORF of *rep* was found downstream of the nonanucleotide motif that was followed by another two ORFs. Again, the accumulation of positively charged aa at the N-terminal end of the putative gene product implies that ORF1 may encode Cp.

The genome length of strain 302_5, 303_5, and 453_5 was 2946, 2946, and 2942 nt, respectively (Figure 2, Table 1). These genomes represented moderate (82.3–82.4%) genome-wide nt identities with the po-circo-like virus 41 strain. The *rep* sequences of the novel genomes showed 100% nt and aa identity with each other and 90.1% nt and 85.8% aa identity with the reference sequence. The putative Cp-encoding ORF1 of strains 302_5, 303_5, and 453_5 showed 100% nt and aa identity with each other and 93.5% nt and 93.7% aa identity with the po-circo-like virus 41 sequence. The ORF2 sequences of the novel genomes shared 99.5–100% nt and 98.5–100% aa identity with each other and 86.5–86.8% nt and 83.6–84.6% aa identity with the po-circo-like virus 41 sequence. Regarding the LIR, the Hungarian porcine origin sequences showed 97.9–99.5% nt identities with each other, and only 56.4–56.8% nt identity with the LIR of the reference genome. The LIR encoded two repeats of 91, 86, and 84 nt long motifs upstream of the TAGTATTAC nonanucleotide motif in the genome of strains 302_5, 303_5, and 453_4, respectively. The function of these, and additional, short repeats along the LIR is unknown.

The other two strains with type V CRESS DNA genome structure, 303_7 and 453_7, had the smallest genomes (2825 nt) and showed 99.9% genome-wide nt identity with each other. However, they shared only 87.3% identity with the genome of the closest reference, the po-circo-like virus 51. The *rep* of the novel sequences from Hungary showed 99.9% nt and 100% aa identity with each other, and 93.2% nt and 93.8 % aa identity with the *rep* of the reference strain. The ORF1 of the two novel strains showed 100% nt and aa identity with each other, and 93.0% nt and 94.3% aa identities with the reference strain sequence. Regarding the ORF2, the two novel strains represented 99.8% nt and 99.5% aa identity with each other, but only 79.9–80.1% nt and 73.6% aa identity with the reference sequence. Similarly, the LIR of the two novel sequences showed 99.6% with each other, and 78.2% with the LIR of the reference genome. Regarding the genome of strain 303_7, the LIR had a highly complex structure; a 52-nt-long region upstream of the nonanucleotide motif (CATTATTAC) consisted of two 34-nt-long overlapping repeats that could be overlaid by three of 16 nt long or six of 6 nt long consecutive repeats. Shorter, 21 nt long, overlapping repeats were detected in the same position within the LIR of strain 453_7 that could be overlaid with two of the 16-nt-long or four of the 6 nt long repeats described for strain 303_7. Of interest, the 16 nt long sequence did not show repeat structure in the LIR of reference sequence. The LIR of 303_7 and 453_7 genomes contained 7 nt long inverted repeats upstream of this complex structure that may imply the formation of loop structure. Downstream of the nonanucleotide motif, 34 nt long repeats were identified in both novel sequences as well, which highly differed from the 38 nt long repeats in the same region of the reference sequence.

### 3.3. Genomic Characterization of Novel FSfaCV-Like CRESS DNA Viruses

The genomes of strains 306_2 and 451_2 were 2921 and 2912 nt long, respectively, and based on the structure of those, could be classified as type II CRESS DNA virus genomes [4] (Figure 2, Table 1). The ORF1 may be encoded on the viral strand downstream of the nonanucleotide motif, while the *rep* may be located in the complementary replicative strand (Figure 2). The sequences showed 98.6% genome-wide nt identity with each other, and 89.2–90.8% nt identities with the genome of reference FSfaCVs. The *rep* of the novel sequences shared 98.8% nt and 98% aa identity with each other, and 90.9–94.9% nt and 87.5–93.4% aa identities with the reference sequences. Accumulation of positively charged amino acids (R and K) in the N-terminal region of the ORF1 indicated that it may code for the Cp. The ORF1 of the novel sequences from Hungary shared 98.3% nt and 98.5% aa identity with each other, and 88.7–90.4% nt and 88.5–89.5% aa identities with the reference sequences. The ORF3 of the novel sequences showed 98.8% nt and 97.5% aa identity with each other, and 92.9–96.5% nt and 86.5–93.9% aa identities with the reference sequences. The ORF2 of strains 306_2 and 451_2 showed 98.4% nt and 96% aa identity among each other; ORF with similar feature could not be identified in the reference genomes. The LIR was located between the 5’ ends of the ORF1 and *rep*. This sequence of the novel FSfaCV genomes showed 99.8% nt identity with each other, and 88.4–92.4% nt identities with the reference sequences. The putative nonanucleotide motif (TAGTATTAC) was surrounded with 10 nt long inverted repeat (GACATAAGGG) that supposed loop formation.

### 3.4. Phylogenetic Classification of the Genomes of Novel Porcine CRESS DNA Viruses

**Figure 3:**
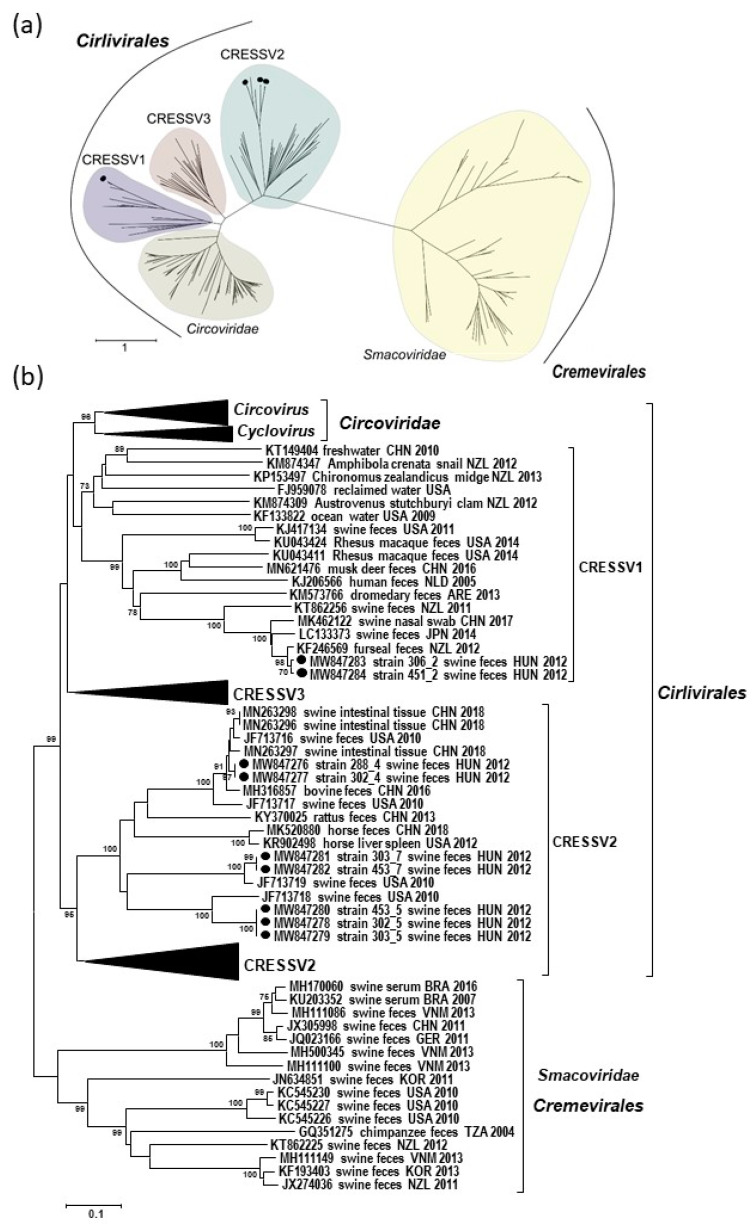
Phylogenetic analysis of the translated *rep* sequences of the viral genomes described in this study. The Hungarian sequences are highlighted with black dots. (**a**) Unrooted maximum likelihood phylogenetic tree generated with the PhyML software, LG + F + G + I model, and aLRT SH-like support, using a subset of reference sequences of the *Cirlivirales* and *Cremevirales* order taken from Krupovic et al. [3]. The scale bar represents the aa substitutions per site. (**b**) Unrooted neighbor joining tree generated with the Mega6 software, p-distance model, and 1000 bootstrap replicates using selected CRESS DNA virus reference sequences. Bootstrap values lower than 70 are not shown. The scale bar represents the aa substitutions per site.

Classification and phylogeny of CRESS DNA viruses is based on the available Rep sequences; thus, to determine the relationship of the novel sequences with other CRESS DNA viruses, we involved this region into phylogenetic analysis using a subset of the reference sequences applied by Krupovic and coworkers [3]. The Rep sequences derived from the genome of different po-circo-like viruses were only distantly related, but grouped together in the phylogenetic tree in a separate branch with unclassified CRESSV2 genomes, including Kirkoviruses (47.1–58.4% nt and 36.2–51.3% aa identities for the *rep*). The Rep sequences derived from the FSfaCVs genome sequences grouped with unclassified CRESSV1 sequences. The FSfaCVs shared as low as 34.5% nt and 20.3% aa identities with the po-circo-like viruses, while the latter showed 47.0–54.4% nt and 37.7–48.6% aa identities with sequences of members from other po-circo-like viral groups. The CRESSV1 and CRESSV2 groups belong to the *Cirlivirales* order together with viruses of the *Circoviridae* family [3] (Figure 3).

## 4. Discussion

In this study, the occurrence of CRESS DNA viruses were investigated in fecal samples of healthy swine collected in a single Hungarian farm. Based on the results of viral metagenomics, the genomic organization of nine viruses from four viral groups were characterized which represented three different genome types [3]. Although the genomic sequence and organization of the novel po-circo-like viruses (strain 288_4, 302_4, 302_5, 303_5, 453_5, 303_7, and 453_7) from Hungary showed marked differences, some similarities were also noted. The rolling circle replication (RCR) mechanism of CRESS DNA viruses is related to conserved Rep motifs, including N-terminal RCR motifs (I, II, and III), C-terminal superfamily 3 helicase motifs (Walker-A, -B, and Motif C) and an arginine finger, that together have a role in initiation, elongation, and termination of RCR of CRESS DNA viruses of eukaryote origin [2]. The Rep motifs identified in the po-circo-like viruses corresponded to that of the circo- and cycloviral genomes (Table 3). On the other hand, the nonanucleotide motif in the LIR of the 288_4, 302_4, 302_5, 303_5, 453_5, 303_7, and 453_7 genomes, featured as NANTATTAC, was also typical for the genomes of viruses belonging to the *Circoviridae* family (Table 1). The motifs were surrounded by inverted repeats denoting potential loop formation that may be necessary for the nicking of the DNA strand at the initiation of RCR [2,4,7]. However, the flanking inverted repeats are generally longer (11 nt long) in the circo- and cycloviral genomes. The LIR of the po-circo-like viruses contained repetitive elements that may help the RCR processes [30].

The putative nonanucleotide motif in the LIR of FSfaCVs genomes was similar to that of po-circo-like viruses and members of the *Circoviridae* family, but the RCR and SF3 helicase motifs of the Rep differed from those (Table 1 and Table 2). In spite of the similarities in the Rep sequences of the po-circo-like viruses, the members of the *Circoviridae* family, as well as those of the FSfaCVs, belonged to separated groups of the *Cirlivirales* order of the CRESS DNA viruses in the phylogenetic trees (Figure 3). In the phylogenetic tree, po-circo-like viruses grouped with CRESSV2, while FSfaCVs clustered with CRESSV1 viruses. Although species demarcation criteria were not defined for CRESSV1 and CRESSV2 viruses, the genetic distance of 302_5, 303_5, and 453_5 (82.3–82.4% nt identity), as well as 303_7 and 453_7 (87.3% nt identity) complete genomes from the reference sequences suggested that those may belong to distinct virus species. The comparisons involving different genomic regions revealed the lowest identities for the LIR. Recombination analysis using reference sequences from the *Cirlivirales* order did not reveal probable recombination events in the sequences of the novel strains (data not shown). However, as CRESS DNA viruses are highly prone to recombination, and the viral genome may consist of sequences originating from the genomes of evolutionary distant taxa of CRESS DNA viruses or from nucleic acid of other viruses, bacteria and plasmid recombination events cannot be ruled out [1,31].

Regarding the putative Cp, N-terminal accumulation of positively charged aa is characteristic for circoviral capsid proteins. This region may be responsible for nuclear localization and for DNA binding during packaging [32]. In the case of the po-circo-like viruses and FSfaCVs, slight accumulation of positively charged aa led us to assign the putative Cp encoding ORFs. However, it is conceivable that gene products of other parts of the genomes may have a role in capsid formation.

Although porcine circoviruses are highly prevalent [9], only traces of *Porcine circovirus 2* sequences could be found in some study samples. Unfortunately, no information was available as to whether the animals were vaccinated against this pathogen in that herd. Besides viruses of the *Cirlivirales* order, smacovirus genomes were also detected in most of our samples. Smacoviruses were identified not only in the feces, but also in serum samples of swine collected in Brazil [20]. Although the low number and quality of the short sequences did not allow more specific analyses, and complete genome sequences were not amplified, smacoviruses seemed to be prevalent in the fecal specimens processed in our study (Figure 1b).

The virome composition of healthy and diseased pigs has been investigated in studies processing fecal, nasal, lymph node, and blood samples collected in numerous geographic areas, including Germany, Sweden, the USA, Brazil, Japan, China, Vietnam, Korea, and New Zealand [10,11,12,13,14,15,16,17,18,19,20,21,33]. However, the number of studies reporting the simultaneous presence of variable CRESS DNA viruses in the same sample is limited [12,14]. Our eukaryotic viral metagenomic sequencing results showed similarities with these earlier studies in that CRESS DNA viral sequences were frequently detected in the fecal virome of the studied Hungarian pigs. This may be the consequence of environmental contamination due to semiclosed husbandry practices. While smacoviruses were described in serum samples that may suppose infection of the affected pigs [20], the po-circo-like viruses and FSfaCVs were feces-associated or originated from intestinal tissue or nasal samples, thus the source of these viruses remains unknown. Although there are differences in the genomic sequences, closely related CRESS DNA viruses of the *Cirlivirales* and *Cremevirales* orders may appear in the intestine of swine and other mammals (e.g., in bovine, fur seal, or primates), regardless of the geographic origin that may imply infection of multiple host species. On the other hand, the CRESS DNA viruses characterized in this study could originate from microbes colonizing the intestinal tract of the hosts, or from other environmental or dietary sources [31,34]. The intense trade of animals and their persistent infection likely contributes to the spread of various viruses that, together with recombination events, may promote emergence of highly diverse viruses and adaptation of those to novel susceptible hosts. Although the pathogenic role of viruses investigated in this study is unknown, which implies that they may be harmless to swine, compliance of the requirements related to animal health and hygiene is of high importance.

## Figures and Tables

**Table 1:** Primer sequences and annealing temperature used for the back-to-back PCR amplification of complete genomes characterized in this study.

Primer Name	Region	Sequenced Genome	Reference Strains	Annealing	Primer Sequences *
CVLV4-F CVLV4-R	ORF1	288_4 302_4	po-circo-like virus 21	53 °C	5’-ATCTTTGGTCTTGCATTGTTGC-3’ 5’-CTTCAAGGCTATCTTATCCTMCC-3’
CVLV5-F CVLV5-R	*rep*	302_5 303_5 453_5	po-circo-like virus 41	58 °C	5’- GACGGTTTTGACCCGTCAACAC-5’ 5’-CCACCACTTGTCAAACGGTTTGAAG-3’
CVLV7-F CVLV7-R	ORF-2	303_7 453_7	po-circo-like virus 51	53 °C	5’-CTGCACCAATAGAAGATGGTAG-3’ 5’-GAGGTTCTGGAATTAAACCATTGTC-3’
CVLV2-F CVLV2-R	*cap*	306_2 451_2	FSfaCVc	53 °C	5’-TAYCTTATGTGGACACATTTACCG-3’ 5’-TAAATTGTGGTTWGGACCATCC-3’

* The primers were designed using the contigs obtained by viral metagenomics.

**Table 2:** The main characteristics, the position, and the length of the ORFs of novel CRESS DNA genomes described in this study.

Genome	Genome Length nt	Genome Type	Nonanucleotide Motif	ORF Position to the Nonanucleotide Motif				
				*rep*	ORF1	ORF2	ORF3	ORF4
288_4	3926	IV	CAGTATTAC	1123–191	1232–1720	1733–2275	2289–2849	3249–2911
302_4	3926	IV	CAGTATTAC	1123–191	1232–1720	1733–2275	2289–2849	3249–2911
302_5	2946	V	TAGTATTAC	226–1203	1366–1941	1956–2543	NA	NA
303_5	2946	V	TAGTATTAC	226–1203	1366–1941	1956–2543	NA	NA
453_5	2942	V	TAGTATTAC	226–1203	1365–1940	1956–2543	NA	NA
303_7	2825	V	CATTATTAC	253–1212	1437–1964	1978–2583	NA	NA
453_7	2825	V	CATTATTAC	253–1212	1437–1964	1978–2583	NA	NA
306_2	2921	II	TAGTATTAT	2478–1423	14–1213	732–1112	2000–1509	NA
451_2	2912	II	TAGTATTAT	2469–1414	14–1204	723–1103	1991–1500	NA

NA; not applicable.

**Table 3:** Endonuclease (rolling circle replication, RCR) and helicase domain motifs of the Rep sequence of the viral strains described in this study.

	RCR Motifs			Superfamily 3 Helicase Motifs		
	I	II	III	Walker-A	Walker-B	Motif C
288_4	CFTIND	PHIQG	YCTK	GKGKS	VIDDW	ITSN
302_4	CFTIND	PHIQG	YCTK	GKGKS	VIDDW	ITSN
302_5	CFTINN	PHIQG	YCSK	GSGKT	VIDDY	VTSN
303_5	CFTINN	PHIQG	YCSK	GSGKT	VIDDY	VTSN
453_5	CFTINN	PHIQG	YCSK	GSGKT	VIDDY	VTSN
303_7	VFTINN	PHIQG	YCSK	GSGKT	LIDDF	ITSN
453_7	VFTINN	PHIQG	YCSK	GSGKT	LIDDF	ITSN
306_2	AMTVKN	QHCHI	YLAK	GSGKS	WFDEF	ISTI
451_2	ALTVKN	QHCHI	YLAK	GSGKS	WFDEF	ISTI

## Data Availability

Sequence data were deposited in GenBank under the following accession numbers: MW847276-MW847284.
